# The ecological consequences of megafaunal loss: giant tortoises and wetland biodiversity

**DOI:** 10.1111/ele.12203

**Published:** 2013-11-25

**Authors:** Cynthia A Froyd, Emily E D Coffey, Willem O Knaap, Jacqueline F N Leeuwen, Alan Tye, Katherine J Willis, Dov Sax

**Affiliations:** 1 Department of Geography, Swansea UniversitySingleton Park, Swansea, SA2 8PP, UK; 2 Department of Zoology, Long-Term Ecology Laboratory, Biodiversity Institute, Oxford Martin School, University of OxfordSouth Parks Road, Oxford, OX1 3PS, UK; 3 Institute of Plant Sciences and Oeschger Centre for Climate Change Research, University of BernAltenbergrain 21, CH-3013, Bern, Switzerland; 4 Charles Darwin Research StationPuerto Ayora, Galápagos, Ecuador; 5 Department of Biology, University of BergenN-5007, Bergen, Norway

**Keywords:** Coprophilous fungi, ecosystem engineer, Galápagos Islands, giant tortoise, megafaunal extinction, wetlands

## Abstract

The giant tortoises of the Galápagos have become greatly depleted since European discovery of the islands in the 16th Century, with populations declining from an estimated 250 000 to between 8000 and 14 000 in the 1970s. Successful tortoise conservation efforts have focused on species recovery, but ecosystem conservation and restoration requires a better understanding of the wider ecological consequences of this drastic reduction in the archipelago's only large native herbivore. We report the first evidence from palaeoecological records of coprophilous fungal spores of the formerly more extensive geographical range of giant tortoises in the highlands of Santa Cruz Island. Upland tortoise populations on Santa Cruz declined 500–700 years ago, likely the result of human impact or possible climatic change. Former freshwater wetlands, a now limited habitat-type, were found to have converted to *Sphagnum* bogs concomitant with tortoise loss, subsequently leading to the decline of several now-rare or extinct plant species.

## Introduction

The Galápagos Islands are globally renowned both for their ecological value and as a world symbol of scientific discovery. An estimated 95% of the native biological diversity of the Islands is still extant (Bensted-Smith *et al*. 2002) making them one of the few remaining options for the maintenance and restoration of a relatively intact archipelago biota, ecosystems which have been severely impacted worldwide. The Galápagos, however, currently exhibit some of the highest extinction rates in the world (Snell *et al*. 2002). This seeming dichotomy is likely the result of the short duration of human presence which did not begin until European discovery in AD 1535.

Significant conservation programmes have been developed in the Galápagos with emphases on three key areas: eradication of invasive species, controlling the introduction and spread of non-native species and preservation of populations of keystone taxa, notably the iconic Galápagos giant tortoise. Since European discovery, Galápagos tortoises have been greatly impacted by people. Tortoise populations throughout the archipelago are estimated to have declined from levels of between 100 000 and 250 000 individuals to a low of 8000–14 000 in the 1970s (Townsend 1925; MacFarland *et al*. 1974), although population numbers have increased in recent years as a result of a successful captive breeding and repatriation programme.

Historically, tortoises were captured by buccaneers and whalers to provide fresh meat aboard ship, hunted for their oil, eaten by settlers and collected during early scientific expeditions. Populations declined further throughout the 19th–20th centuries with the impacts of introduced non-native animal species. Of the 14 generally recognised Galápagos tortoise taxa (Poulakakis *et al*. 2012), four became extinct during the historical period (i.e. since AD 1535, but prior to the development of modern conservation programmes) and another in 2012 with the death of Lonesome George, the only surviving Pinta Island tortoise (although see Edwards *et al*. 2013); the remaining nine taxa are all presently threatened with extinction (IUCN).

While preservation of the giant tortoise species themselves has been a conservation focus, the functional role that giant tortoises may have played on island systems globally is increasingly recognised (Griffiths *et al*. 2010; Hunter *et al*. 2013; Pedrono *et al*. 2013). Successful conservation and ecosystem restoration in the Galápagos requires an understanding of the baseline conditions prior to human arrival and the drivers behind native species loss, but there is still little knowledge of the full extent of the ecological impacts and trajectories that may have been set in motion by such a drastic reduction in this keystone species. Tortoises are known ecosystem engineers, physically modifying their habitats through grazing, seed dispersal, trampling, impacts on nutrient cycling and by altering vegetation composition through selective herbivory and the creation of canopy openings for light-dependent plant species (Gibbs *et al*. 2010; Griffiths *et al*. 2011; Blake *et al*. 2012). Their functional role may be amplified in the Galápagos, where as the largest native terrestrial herbivore, giant tortoises likely played a key role in ecosystem development (Hansen & Galetti 2009; Hansen *et al*. 2010).

The introduction of non-native extant tortoises to restore missing ecological functions has been proposed to mitigate for the ecological consequences of giant tortoise extinction on islands in the Galápagos, the Mascarenes and Madagascar (Griffiths *et al*. 2010; Hunter *et al*. 2013; Pedrono *et al*. 2013). However, there is still a significant knowledge gap as to the full extent of the impact that tortoise population loss has had on island ecosystems. Impacts that must be considered include not only complete extinctions but also effects on ecosystem composition and function on islands still supporting wild tortoises, but with significantly reduced populations.

A key question is whether population reduction led to changes in either the geographical distribution of giant tortoises or their habitat usage, and what impacts such changes may have had on island ecosystems. Of particular interest is to what extent, if any, tortoises may have formerly utilised high elevation inland habitats on some of the larger islands of the Galápagos where their presence has never been documented. The most extensive wild tortoise populations in the islands today are found on the shield volcanoes of Isabela Island, which reach up to 1100–1700 m in elevation. Tortoises, however, do not presently occupy high elevations on the other islands and it is not known to what extent they may have used these habitats in the past. Early accounts indicate that tortoises were abundant in coastal regions during the wet season, moving inland during the dry season in search of water (Townsend 1925). Charles Darwin (Darwin 1845) described the network of tortoise trails on San Cristóbal Island and long-distance travel by tortoises to highland water sources. Historical distributions are particularly interesting on the central island of Santa Cruz, the most developed island in the archipelago, where over 86% of highland habitats are presently classified as degraded (Watson *et al*. 2010). Santa Cruz supports what is currently the second largest number of wild tortoises in the Galápagos, occupying both arid lowland and more humid mid-elevation habitats where they congregate around seasonal wetlands for drinking and wallowing. Recent GPS tracking studies confirm long-distance altitudinal migration by tortoises on Santa Cruz, travelling linear distances > 10 km and reaching elevations of 423 m a.s.l. (above sea level) (Blake *et al*. 2013).

We used palaeoecological records of coprophilous (dung-affiliated) fungal spores, an indicator of large herbivore presence (Burney *et al*. 2003; Davis & Shafer 2006; Gill *et al*. 2009), pollen, plant fragments and charcoal over the last 5500 years to examine: (1) Whether there is evidence that tortoises were formerly present at elevations on Santa Cruz Island above their present range [i.e. in the Humid zone above *c*.420 m a.s.l. (Tye & Francisco-Ortega 2011)], (2) If so, the ecological impacts of tortoise population loss from upland ecosystems and (3) Potential factors contributing to tortoise population decline and range contraction.

## Materials and Methods

### Site location

Sediment cores were collected from three *Sphagnum* bogs, designated ‘East’, ‘Psidium’ and ‘Pernettya’ Bog (Appendix 1*,* Fig. S1, Table S1), located within volcanic craters in the high elevation fern–sedge vegetation formation (570–864 m a.s.l.) of the Humid zone (Tye & Francisco-Ortega 2011). A seasonal atmospheric inversion layer forming between June and December creates ground-level semi-permanent misty conditions in the uplands of the larger, higher islands, a phenomenon known locally as garúa. This has allowed the development of lush, humid vegetation communities including, on Santa Cruz, fourteen identified raised bogs (Itow & Weber 1974) (Appendix 1*,* Fig. S1). Sediment samples were also collected from seasonal wetlands at El Chato, Santa Cruz Island (S 0° 40' 21”, W 90° 26' 18”), a mid-elevation site (200 m) presently utilised by tortoises. Impenetrable gravel layers and the effects of seasonal drying precluded the recovery of a full sedimentary sequence, but the site was used as a control to evaluate evidence of tortoise presence within the highland palaeoecological records.

### Fossil pollen, spore, non-pollen palynomorph, charcoal and macrofossil analyses

Fossil pollen, spores, non-pollen palynomorphs (including spores of coprophilous fungi) and microfossil charcoal (< 180 μm) were analysed for each of the sedimentary sequences following standard methodologies (Bennett & Willis 2001; van Geel 2001; Finsinger & Tinner 2005), see Appendix 1. The cores were sampled at 4 cm intervals in the East and Psidium Bog sequences and 2 cm for Pernettya Bog. Palynomorph concentrations per cm^3^ of sediment were calculated for each taxon over time. Relative abundance (%) of *Sporormiella* and charcoal was calculated as a proportion of the individual sum added to the total land pollen sum (ΣTLP). Macrofossil abundance (per 50 cm^3^ of sediment), i.e. preserved plant fragments, such as seeds, flowers, leaves, roots and wood > 125 μm, were assessed from East and Pernettya bogs following Birks (2001).

### Dating

Twenty-six samples were radiocarbon dated to determine the ages of the sedimentary sequences (Appendix 1, Table S2). Linear age–depth models were used to interpolate sediment ages (Appendix 1*,* Figs S2–S4). The uppermost 17 cm of the East Bog sequence was dated using ^210^Pb (Appendix 1*,* Table S3). Ages are reported as years before present, where ‘present’ is AD 2005, the year the sediment cores were collected.

## Results

### Coprophilous fungi

**Figure 1:**
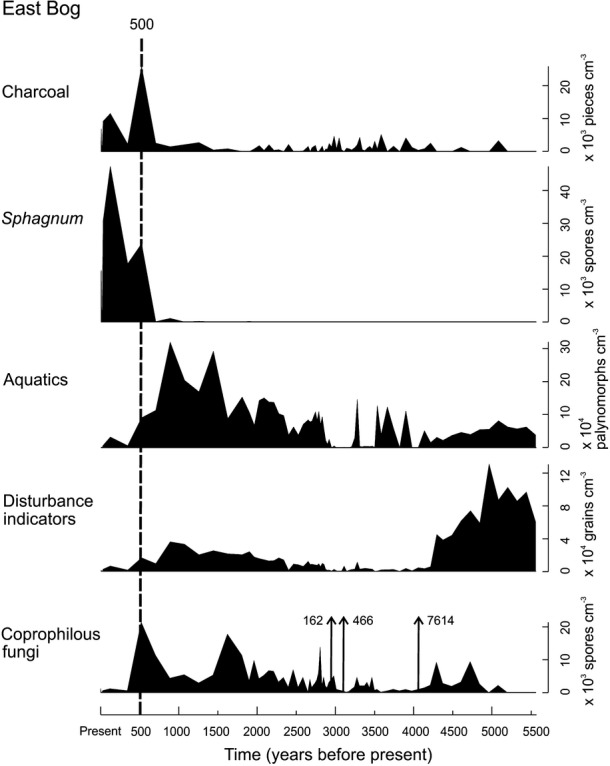
Variation in concentration (per cm^3^ of sediment) of selected pollen and spore types and microscopic charcoal over time at East Bog (see also Appendix S1, Figs S5, S6). Ages are displayed as years before present, where Present = AD 2005. Aquatic taxa include the following: *Utricularia foliosa*, *Azolla microphylla*, *Botryococcus* and *Riccia*. ‘Disturbance indicators’ are plant species which occupy disturbed, muddy environments and likely indicative of the impacts of tortoise wallowing. These include the following: *Ageratum conyzoides, Borreria dispersa* species complex*, Spermacoce remota, Commelina diffusa, Cuphea carthagenensis, Drymaria cordata*-type*, Jaegeria gracilis, Ludwigia erecta*-type*, Phyllanthus carolinianus, Polygonum* Sect. *Persicaria* and *Ranunculus flagelliformis*. Coprophilous fungi include: *Sporormiella, Cercophora* (2 species) and *Podospora*.

**Figure 2:**
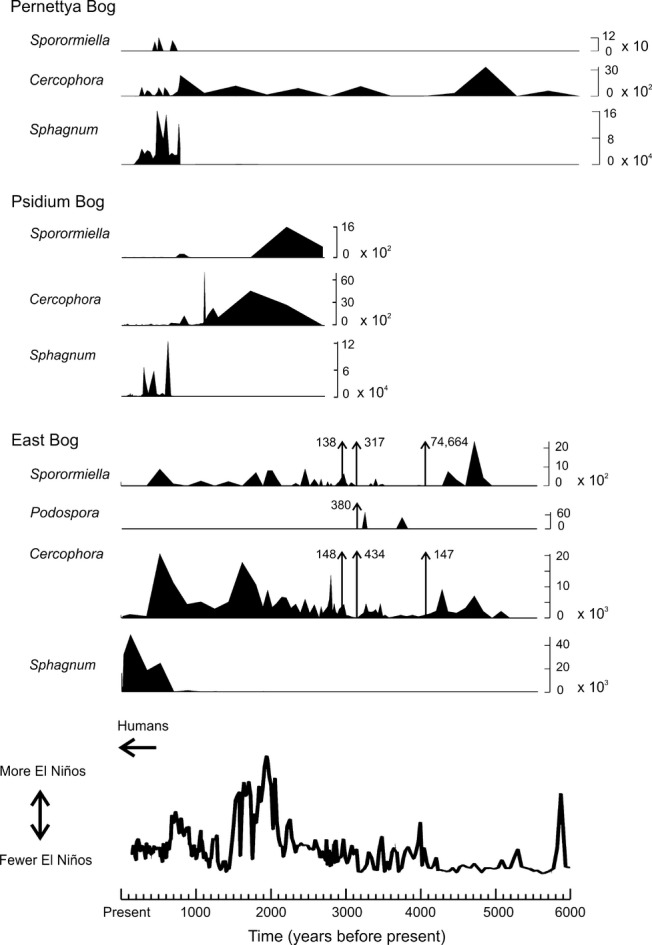
Variation in concentration (per cm^3^ of sediment) of spores of coprophilous fungi over time at Pernettya, Psidium and East Bog. The timing of expansion of *Sphagnum* at each site, as well as possible drivers behind tortoise loss, is also illustrated. The first known human presence in the Galápagos archipelago occurs at AD 1535. The El Niño frequency curve for the Galápagos is modified from Conroy *et. al*. (2008) and is based on proxy records of % sand occurrence in lake sediments from El Junco lake, San Cristóbal Island; post-AD 1850 data have been excluded as a result of likely anthropogenic impact on the record (Conroy *et al*. 2010).

All three sedimentary sequences contain coprophilous fungal spores, indicating the former presence of grazing herbivores in the highlands of Santa Cruz Island. Palynological results reveal that abundance of coprophilous fungi declined with the expansion of *Sphagnum*, occurring approximately contemporaneously within the temporal sampling resolution of the sediments (Fig. 1; see also Appendix 2*,* Figs S5, S6). Abundant spores of the coprophilous fungi *Sporormiella* and *Cercophora* (2 species) were found at all three sites, as well as *Podospora* at East Bog (Fig. 2), revealing the consistent presence of dung-affiliated fungi for over 5000 years, prior to the recent period of *Sphagnum* development (last *c*.500–700 years). There are also three large peaks in coprophilous spore abundance in the East Bog sequence (Fig. 2) between 3000 and 4000 years ago, evidence of high dung concentrations at the site of coring. Detailed examination of these sediment samples revealed the presence of nine additional coprophilous fungal taxa: *Fimetariella*, *Sordaria*, *Coniochaeta* (2 species), *Hypocopra* (2 species), *Delitschia*, *Trichodelitschia* and *Petriella*. Anomalously high concentrations (up to 200 times greater) of pollen of some herbaceous plants including *Acalypha, Amaranthus, Evolvulus*, *Lantana peduncularis* and *Pilea baurii* are also associated with the dung-rich samples, likely indicating pollen input of forage species via the dung of grazing herbivores.

**Figure 3:**
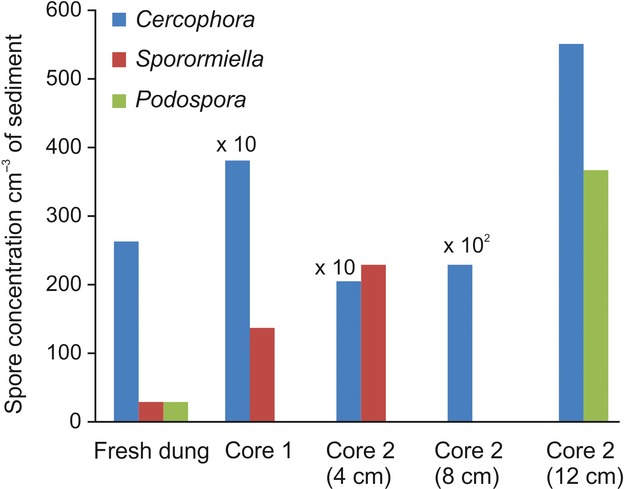
Coprophilous fungal spore concentrations (per cm^3^ of sediment) from present-day giant tortoise habitats at El Chato, Santa Cruz Island. Diagram shows spore abundance from: (1) Fresh tortoise dung collected from the ground surface and cultured in the laboratory, (2) A sediment sample taken from 28 cm deep within the modern sediments (Core 1), (3) Sequential samples analysed from a second sedimentary core (Core 2) at depths of 4, 8 and 12 cm, all of which radiocarbon dated as modern (i.e. post-AD 1950) (Table S2).

Dung from giant tortoises, the only large native herbivore in the Galápagos, was cultured in the laboratory. Coprophilous fungal spores were analysed from the dung as well as from modern sedimentary material [radiocarbon dated as post-AD 1950 (Appendix 1*,* Table S2)] from ephemeral ponds in present-day tortoise habitat at El Chato, Santa Cruz (Fig. 3; Appendix 2, Fig. S7). Patterns of spore abundance are similar to those observed in the highland palaeoecological records, containing abundant *Cercophora* spores, as well as the presence of *Sporormiella* and *Podospora*.

Coprophilous fungi are commonly accepted as a reliable indicator of large herbivore presence (Burney *et al*. 2003; Davis & Shafer 2006; Gill *et al*. 2009; Rule *et al*. 2012; Baker *et al*. 2013), although see Wood & Wilmshurst (2012). The East Bog sequence provides the strongest evidence of past large herbivore occupation of the uplands, evidence which is supported by the other two sites. *Podospora* and particularly *Sporormiella*, both obligate coprophiles (Baker *et al*. 2013), are abundant throughout the East Bog sequence (Fig. 2) prior to *Sphagnum* expansion at 500 ± 20 cal. BP (Fig. 2; Appendix 1, Table S2), with abundant *Cercophora*, a common coprophile, fluctuating in parallel. Although dung spore concentrations, as presented here, have been shown to provide the most accurate measure of grazing intensity (Gill *et al*. 2013), determination of large herbivore presence has traditionally been based on minimum percentage threshold occurrence values of *Sporormiella* alone (calculated as a proportion of the pollen sum), conventionally 1 or 2% (Baker *et al*. 2013; Gill *et al*. 2013). Relative abundance of *Sporormiella* at East Bog consistently exceeds the 2% threshold, reaching 76–99% in the coprophile peaks (Appendix 2, Table S4). This, coupled with ten additional dung-affiliated spore types discovered at the site, provides compelling evidence for past abundance of grazing herbivores in the Santa Cruz highlands. *Sporormiella* is less abundant in the other two sequences, but high concentrations of *Cercophora* spores at Pernettya and Psidium Bog follow the pattern at East Bog – consistent presence prior to the development of *Sphagnum*. Relative abundances of *Sporormiella* at Psidium Bog are commonly 1 – 2% (Appendix 2, Table S4), with minimal occurrence at Pernettya Bog. The species abundance ratio amongst spore types is consistent with data obtained from modern tortoise habitat, both in sediments and growing on fresh dung (Fig. 3), which show *Cercophora* spores to be more abundant than *Sporormiella*. Potential contamination of the coprophilous fungi record as a result of down-washing of modern spores or impacts of increased fungal activity (Wood & Wilmshurst 2012) can be clearly discounted within these sequences (see Appendix 2, Fig. S8).

### Wetland habitats

The pollen results indicate significant habitat transitions occurring in the Galápagos highlands over the last 5500 years (Fig. 1; see also Appendix 2*,* Figs S5, S6). The present-day *Sphagnum* bog ecosystems are revealed to be relatively recent, developing only during the last 500–700 years and replacing former wetland habitats. Prior to 500 ± 20 cal. BP, the East Bog site (Fig. 1) supported a freshwater pond. Pollen of aquatic taxa including the plants *Utricularia foliosa*, *Azolla microphylla* and *Riccia*, as well as the green alga *Botryococcus*, are abundant in the fossil pollen record. Macrofossil analysis (Coffey 2012) revealed five additional wetland taxa to be present during the period preceding *Sphagnum* expansion, including *Potamogeton pectinatus, Ruppia maritima,* the alga *Nitella*, the cladoceran *Ceriodaphnia* sp. *cf. Ephippium* and the aquatic plant *Elatine* sp. (Appendix 2*,* Fig. S9), which is the first reported occurrence of this genus in the Galápagos. The other two analysis sites, Psidium and Pernettya Bog (Appendix 2, Figs S5, S6) show similar habitat transitions, from open water or Cyperaceae-dominated freshwater wetlands to *Sphagnum* bogs, although *Sphagnum* development appears to have occurred earlier at these sites, 560 ± 55 cal. BP at Psidium Bog and 740 ± 55 cal. BP at Pernettya Bog (Appendix 1*,* Table S2). In addition to aquatic taxa, other species declined in synchrony with this habitat transition, including plants indicative of disturbed, muddy environments such as *Ageratum conyzoides, Borreria dispersa* species complex*, Spermacoce remota, Commelina diffusa, Cuphea carthagenensis, Drymaria cordata*-type*, Jaegeria gracilis, Ludwigia erecta*-type*, Phyllanthus carolinianus, Polygonum* Sect. *Persicaria* and *Ranunculus flagelliformis*.

### Rare plants

**Figure 4:**
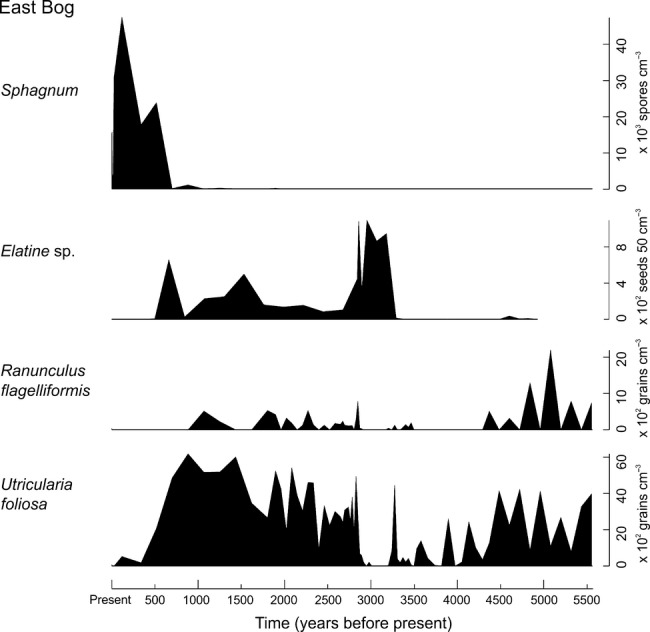
Variation in selected rare plant species and *Sphagnum* over time at East Bog. *Utricularia foliosa*, *Ranunculus flagelliformis* and *Sphagnum* are pollen/spore concentration per cm^3^ of sediment. *Elatine* sp. curve shows concentration of macrofossil seed fragments > 125 μm per 50 cm^3^ of sediment and were analysed from an adjacent, independently dated sedimentary sequence.

The transition from wetland to bog corresponds with the local extinction or decline of several now-rare plant species in the Galápagos. Fossil seeds of *Elatine* sp., an aquatic plant no longer present in the archipelago today, occur throughout the former wetland periods at both East and Pernettya Bog (Fig. 4). Macrofossils from Psidium Bog were not analysed in this study. It is not yet known whether the Galápagos *Elatine* sp. is a mainland species or new to science. Genetic analyses are required to make this determination. In addition to the (at least local) extinction of *Elatine*, loss of open water habitat also led to the reduction of other native wetland-dependant plants (Fig. 4), now rare in the Galápagos, including *Ranunculus flagelliformis* and *Utricularia foliosa* (Adsersen 1989; van Leeuwen *et al*. 2008).

### Charcoal

**Figure 5:**
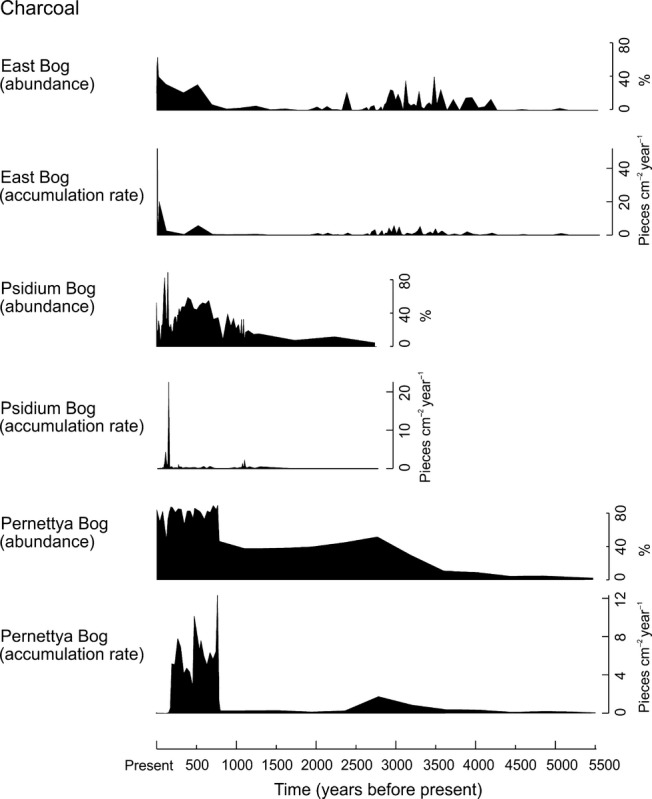
Variation in abundance (as % of total pollen sum) and accumulation rates (pieces cm^−2^ year^−1^) of charcoal fragments >10 μm and <180 μm over time at East Bog (*c*.5500 years) and Psidium Bog (*c*.1200 years) and >10 μm and <250 μm at Pernettya Bog (*c*.5500 years).

Charcoal abundance increased at all three analysis sites concurrently with wetland loss and *Sphagnum* development (Figs 1 and 5). Charcoal results (Fig. 5) are displayed both as relative abundance (%) (van der Knaap *et al*. 2012) and as accumulation rates over time (pieces > 10 μm cm^−2^ year^−1^). Charcoal accumulation rates, particularly at Psidium and Pernettya Bog, may be influenced by changes in sedimentation rates and faster growing peat in the upper sections of the sequences (Appendix 1, Figs S3, S4). Both measures indicate increasing fire across the highlands. Other fire indicators found to increase concurrently with bog expansion include *Pteridium* (van der Knaap *et al*. 2012) and the fungus *Gelasinospora* (Appendix 2*,* Fig. S8).

## Discussion

### Giant tortoises in the Santa Cruz highlands

The high concentrations of coprophilous fungal spores discovered in the sedimentary sequences, with their very limited dispersal distances (Davis & Shafer 2006; Raper & Bush 2009; Gill *et al*. 2013) and the enclosed nature of the cinder cone basins examined, provide evidence of the former local presence of large herbivores in the Santa Cruz highlands – likely the giant tortoise, the archipelago's only large native herbivore. The only other moderately large animal species conceivably capable of depositing abundant quantities of dung is the extinct giant rice rat, *Megaoryzomys* sp. (Steadman *et al*. 1991). It is unclear, however, whether this species would have used these wetland habitats to such an extent, whereas this is consistent with the known habitat preferences of the giant tortoises (Rodhouse *et al*. 1975; Appendix 2, Fig. S10) which presently utilise seasonal mid-elevation (*c*.200 m) ponds on Santa Cruz and higher altitude ponds on Isabela Island for drinking, grazing and wallowing for protection from parasites and, being ectothermic, to regulate their body temperature. Input of faecal matter from birds, another potential although unlikely (Wood *et al*. 2011; W. Gams, pers. comm.) coprophile producer, would not have been a factor in the Galápagos which supports no common anatids or significant populations of avian taxa favouring upland freshwater habitats (Appendix 2, Table S5).

Pollen of herbaceous taxa of disturbed, muddy environments was found to coincide with the presence of coprophilous fungi, providing further evidence of past tortoise occupation (Fig. 1 ‘Disturbance Indicators’, see also Appendix 2, Figs S5, S6). These species indicate environments that would be created through the physical impacts of tortoise trampling and wallowing, which would have helped maintain open, muddy conditions. Wallowing activity by bison in the tallgrass prairies of North America, for example, has been shown to increase the abundance of disturbance-related plants (Trager *et al*. 2004). Additionally, tortoises would have impeded the development of *Sphagnum*, a known habitat modifier (van Breemen 1995), through both the physical disturbance of wallowing as well as by direct grazing of *Sphagnum* plants (Rodhouse *et al*. 1975).

The evidence provided in this study therefore comprises the first record of likely former tortoise occupation within the high elevation (up to 800 m) fern–sedge zone on Santa Cruz Island, well above the known present-day migrational limits of *c*.400 m (Blake *et al*. 2013). The dung spores declined from the fossil record at East Bog (Fig. 1) around 500 ± 20 cal. BP, indicating loss of tortoises from this time onwards and coinciding with a decline of open water habitat and subsequent development of the present-day *Sphagnum* bog ecosystem. Similar transitions occurred at the other two analysis sites between 500–700 years ago.

### Ecological consequences of tortoise loss

These results suggest that former occupation of the Santa Cruz uplands by giant tortoises may have been intrinsically linked with the presence of historical freshwater wetlands, a now-rare ecosystem type in the Galápagos providing both biotic and landscape-level diversity. Freshwater habitats in the Galápagos are presently restricted to: El Junco Lake on San Cristóbal Island, the only permanent freshwater body in the archipelago, and a limited number of springs and ephemeral ponds, most notably El Chato on Santa Cruz and seasonal ponds on Isabela Island where tortoises presently congregate.

Worldwide, research on the extinction of megafauna has focused primarily on causation while less attention has been paid to the resultant long-term ecological consequences (Rule *et al*. 2012). In a system such as the Galápagos Islands, the loss of a solitary ‘island giant’ may have a relatively greater functional impact than the better known Pleistocene megafaunal extinctions occurring on continents (Hansen & Galetti 2009). The habitat-shaping effects of tortoise grazing and seed dispersal have been reported (Gibbs *et al*. 2010; Griffiths *et al*. 2011; Blake *et al*. 2012), but the key functional role that tortoises may have played in the long-term maintenance of wetland communities through the physical impacts of wallowing has not previously been identified.

The loss of narrowly distributed open water habitat through encroachment of vegetation as a direct consequence of the removal of grazing herbivores has been demonstrated in desert spring areas in both the United States and Australia (Kodric-Brown & Brown 2007). We suggest similar ecological dynamics occurring in the Galápagos – that the giant tortoises likely functioned as ecological engineers in upland systems, maintaining freshwater wetlands and the native biodiversity they supported. The importance of the physical impacts of wallowing in maintaining habitat for wetland species has previously been identified for example in bison wallows in the Great Plains of North America (Collins & Uno 1983). While we suggest the tortoises themselves as the primary ecological driver, the possibility that climatic factors (i.e. through periodic drying of the wetlands or some other factor limiting tortoise occupation in the highlands) played a role cannot be discounted, although the observed habitat transitions to the present *Sphagnum*-dominated ecosystems are quite rapid to be purely climatic in origin. Coprophilous fungal spore concentrations were found to be variable over time, a common occurrence within palaeoecological records likely resulting from a combination of factors including spore depositional environments, sedimentation and herbivore behaviour, although this may also potentially be indicative of fluctuating tortoise population abundance in response to possible climatic shifts. Fluctuations in aquatic species abundance and coprophilous fungi appear to be particularly dynamic at East Bog between 3000 and 4000 years ago (Fig. 1), possibly indicating episodic drying. Interestingly, although this period has some of the lowest background levels of coprophilous spores, it also includes large peaks of exceptionally high abundance of dung-affiliated fungi.

Conversion of the former freshwater wetlands to *Sphagnum* bogs led to the extinction or decline of several rare plant species. Fossil seeds of *Elatine* sp., an aquatic plant which has not been reported in the Galápagos before, were found throughout the former open water wetland periods at both East and Pernettya Bogs (Fig. 4). It appears to have gone extinct in the archipelago with the loss of these wetland habitats. In addition to the local extinction of *Elatine*, loss of open water habitat also led to the reduction of other native wetland-dependant plants (Fig. 5), now rare in the Galápagos, including *Ranunculus flagelliformis* and *Utricularia foliosa* (Adsersen 1989; van Leeuwen *et al*. 2008), the latter now known only from El Junco Lake on San Cristóbal Island. Schofield & Colinvaux (1969) similarly report the extinction of an aquatic fern, *Azolla filiculoides*, associated with a Pleistocene drying event at El Junco. In the Galápagos where to date only three plant species' extinctions have been recorded since human arrival (Tye 2000), the loss of restricted habitat types such as the former upland wetlands reported here may have significant impacts on native biological diversity, not only of plants but of other aquatic organisms.

Loss of the tortoises and wetland habitats may have also led to impacts on larger landscape-level ecosystem processes including hydrological function on the water-limited island of Santa Cruz, as well as potential changes in upland fire regimes. Increasing charcoal abundance (Fig. 5) was found to have occurred concomitant with the evidence of tortoise decline (Figs 1 and 5). Enhanced fire regimes are a reported consequence of large herbivore extinction worldwide, including following the loss of giant tortoises in Madagascar, as a result of increases in flammable biomass due to reduced grazing (Burney *et al*. 2003; Gill *et al*. 2009; Rule *et al*. 2012; Pedrono *et al*. 2013). The increases in charcoal (Fig. 5) as well as other fire indicators, i.e. bracken and the fungus *Gelasinospora*, observed in the sedimentary sequences may be indicative of the same process occurring in the Galápagos. Griffiths *et al*. (2010) describe tortoise extinctions in Mauritius as having left a ‘legacy of ecosystem dysfunction’. Similarly, the loss of giant tortoises from the Galápagos highlands has altered ecological trajectories, the effects of which may not yet be fully apparent given the relatively short period since impact.

### Potential drivers of tortoise decline and wetland loss in the highlands

These dramatic habitat shifts were the consequence, but what caused tortoise populations to decline in the highlands? Possible explanations include both human agency and climatic change, and it is likely that a combination of these factors contributed to tortoise loss. Humans are known to have severely impacted tortoise populations, both directly and indirectly, throughout the archipelago. Our results reveal a clear increase in charcoal, a common indicator of anthropogenic presence, throughout the historical (post-European discovery) period (Fig. 1). Although Santa Cruz was not permanently inhabited until the early 19th century, this suggests that early post-discovery human impact on Santa Cruz may potentially have been greater than has been believed. The increase in charcoal occurring contemporaneously with declining fungal spore abundance (Fig. 1) suggests human-induced extirpation as a potential driver behind tortoise population decline and subsequent wetland conversion. Although conversely, the increased charcoal may instead itself be a consequence of tortoise population decline, the result of increased fire due to reductions in grazing.

The timing may also be too early to confidently implicate human agency as the primary driver. The Galápagos Islands were first discovered by Europeans in AD 1535 and there is no firm evidence of earlier human presence. While the age determination for the observed habitat shift at East Bog occurs slightly earlier than this (500 ± 20 cal. BP), given the inherent imprecision in radiocarbon dating and the resolution of the sedimentary samples, the decline in dung spores and the *Sphagnum* transition could be reasonably interpreted as being temporally concurrent with early human presence. This transition, however, appears to have begun earlier at the other two sites examined: Psidium Bog (560 ± 55 cal. BP) and Pernettya Bog (740 ± 55 cal. BP) (Fig. 2), although Pernettya Bog is the least securely dated of the three sequences (Appendix 1, Table S2).

Historically, tortoise populations throughout the Galápagos were significantly depleted through hunting. Immediately post-discovery, however, human visitation to the Islands remained transient and it seems unlikely, with plentiful tortoise populations at lower elevations, that early visitors would have penetrated the dense interior and highest reaches of Santa Cruz purely for hunting purposes, although use of the former ponds as a freshwater source with consequent incidental hunting is a possible explanation. Alternatively, it may be that reductions in coastal populations, both directly by hunting and through predation on tortoise eggs by introduced rats, led to reduced competition for resources in the lowlands and meant that tortoises no longer needed to migrate to the highlands. This is supported by the forage-driven seasonal altitudinal migration patterns of present-day giant tortoise populations on Santa Cruz (Blake *et al*. 2013). Reduction in migration of tortoises into the highlands could also account for a time-transgressive pattern of bog development.

An alternative possible driver behind tortoise population decline is a climatic shift, either directly affecting the tortoises or their wetland habitats. A factor that may presently limit tortoise habitation in the uplands of Santa Cruz is the seasonal garúa mist, which creates cool conditions that may be beyond the thermoregulatory capacity of the species (J.P. Gibbs, pers. comm.). Galápagos tortoise populations presently occupy high elevation habitats (> 1000 m) on northern Isabela Island, but conditions are not analogous as these sites are generally much drier than the fern–sedge zone habitats on Santa Cruz. Little, however, is known about the long-term climatic history of this seasonal inversion phenomenon. Presently, during El Niño years, the cool season is reduced and more tropical climatic conditions prevail throughout the archipelago (Trueman & d'Ozouville 2010). Palaeoclimate reconstructions indicate higher El Niño frequency in the Galápagos prior to AD 1400 (Conroy *et al*. 2008; Sachs *et al*. 2009) (Fig. 2), suggesting a corresponding reduction in cool season conditions. It is therefore possible that the high elevation garúa mists were less prevalent in the past, either forming less often, for shorter periods on either a daily or seasonal basis, or were even completely absent (Bush *et al*. 2010). Tortoise population declines may have therefore resulted from the inception of the present-day climate regime.

It is difficult to disentangle the impacts that changing climatic conditions may have had on upland wetlands. Presently, during El Niño years the islands receive more rainfall, however garúa conditions, which provide moisture input and limit evaporation in the highlands are reduced, and temperatures are generally higher. Changing conditions either in terms of temperature or effective moisture could potentially have influenced the tortoises either directly or via effects on their wetland habitats. Apart from the reduction in El Niño frequency and variability (Conroy *et al*. 2008) (Fig. 2) palaeoclimate reconstructions for the Galápagos uplands do not reveal any distinct changes at the time of bog inception and wetland loss on Santa Cruz (500–700 years ago). Drought-induced drying of the wetlands could potentially have precipitated conversion to *Sphagnum*. However, palaeoclimate records obtained from El Junco Lake on San Cristóbal Island, an area analogous to highland conditions on Santa Cruz, do not indicate climatic drying over the period in question. Sachs *et al*. (2009), based on analyses of botryococcene δD, estimate that conditions in the Galápagos remained wet throughout the period of the observed wetland transition on Santa Cruz, only becoming drier following AD 1830, and analyses of sedimentary grain size (% clay and silt) by Conroy *et al*. (2008) indicate that the period *c*.500–700 cal. BP had some of the wettest background climate conditions from throughout the Holocene. While it is likely that climate played a role in the habitat transformations, there is no clear evidence from existing palaeoecological reconstructions to indicate significant drying in the Galápagos uplands as the driver behind the observed rapid change in wetland habitat.

A combination of factors, i.e. potential climatic changes exacerbated by changes in tortoise migration, therefore is the most likely explanation to account for the functional extinction of giant tortoises from the Santa Cruz highlands. Our results add to growing evidence that, far from being the ‘pristine ecosystems’ of common perception, the Galápagos Islands, despite uniquely high rates of native species retention, have experienced significant habitat transformations. The Galápagos *Sphagnum* crater bogs are found to be a relatively recent development, replacing former open water habitats likely as a consequence of the loss of tortoises from the highlands. This has important conservation implications both for the species and more widely, in terms of ecosystem restoration and conservation.

Giant tortoises were once globally distributed, but today survive in the wild only in the Galápagos and on Aldabra in the Indian Ocean (Hansen *et al*. 2010). In a number of island systems, including the Mascarenes, Seychelles, Madagascar and some islands in the Galápagos, tortoise population extinctions have occurred relatively recently as a result of human occupation, so the full impact of their loss may not yet be apparent. Recognition of the key ecosystem role of giant tortoises has led to the development of re-wilding programmes to introduce non-native extant tortoises in order to restore missing ecological functions (Griffiths *et al*. 2011; Hunter *et al*. 2013; Pedrono *et al*. 2013). A better understanding of the functional role that tortoises played historically in the Galápagos can only serve to enhance conservation both in the Galápagos and in other former tortoise-occupied habitats worldwide. These findings support growing evidence of the extent of the ecological consequences of the extinction of large herbivores globally (Gill *et al*. 2009; Smith *et al*. 2010; Rule *et al*. 2012) and identify an aspect that is often not considered – the effect of megafaunal loss on specialised wetland habitats and the unique organisms and ecosystem functions they maintained.
