# Supplementary material for: The ecological consequences of megafaunal loss: giant tortoises and wetland biodiversity
Source: Ecol Lett. 2013 Nov 25;17(2):144–54. doi: 10.1111/ele.12203 (PMC4015371; doi:10.1111/ele.12203)
Supplement: Supplementary file 1 [file ele0017-0144-sd1.pdf]

Supporting Information

Appendix 1

Materials and Methods

Site description and sampling methodology

Sediment cores were collected from the center of the three *Sphagnum* bogs using either a 5cm-diameter *GeoCore* piston corer (East and Psidium Bog pollen cores, Pernettya Bog macrofossil core) or a gouge corer (Pernettya Bog pollen core). East and Psidium Bog are approximately 1.1 km apart, situated within cinder cone craters that form part of the east–west orientated Cerro Crocker central ridge system of the island. The Pernettya Bog site is an isolated cinder cone crater, approximately 0.7 km south of Cerro Crocker. Pernettya and Psidium Bog are within steep-sided, enclosed basins approximately 30 m deep, while the East Bog basin is shallower with crater walls approximately 10 m in height.

Fossil pollen, spore, non-pollen palynomorph and charcoal analyses

Sediment cores were extruded from the corer in the field, wrapped in plastic film, aluminum foil and thick plastic, transported back to the laboratory and stored in the dark at 4° C. The cores were analysed for fossil pollen, spore, non-pollen palynomorph, including coprophilous fungi, and charcoal content. Samples (0.56 cm^3 in volume) were taken at 4cm intervals in the East and Psidium Bog sequences. Larger sample volumes were analysed from the upper segments of the sequences which were composed of fresh *Sphagnum* (3.36 cm^3 in the topmost 29 cm at East Bog; 1.68 cm^3 in the upper 152 cm at Psidium Bog). Samples (1.0 cm^3 in volume) were taken at 2 cm intervals in the Pernettya sequence, with larger sample volumes (8–20 cm^3) in the topmost 10 cm. The temporal sampling resolution over the last 500 years was 77 years at East Bog, 10–37 years at Psidium Bog and 35 years at Pernettya Bog.

Fossil palynomorph extraction from the sedimentary material followed standard processing methodology (Bennett & Willis 2001). Sediments were treated with hydrochloric acid for the removal of carbonates and either hot sodium hydroxide followed by coarse sieving at 180 μm (East, Psidium Bog and El Chato) or hot potassium hydroxide followed by coarse sieving at 250 μm (Pernettya Bog), to remove humic acids and bulk material. Samples were treated with hydrofluoric acid for the removal of silica and silicates and acetolysis to remove

polysaccharides. A known quantity of exotic *Lycopodium* spores was added to each sample for the determination of pollen concentrations (Stockmarr 1971). Pollen, spore, non-pollen palynomorph (van Geel 2001) and microfossil charcoal abundance (Finsinger & Tinner 2005) were tallied at each sampling level using a transmitted light microscope at x400 magnification. A minimum of 400 identifiable pollen grains and spores of vascular plants (excluding exotic markers) were counted for each sample, up to a maximum of 65,690 for samples with high fern spore counts. The ANSI C program Psimpoll (4.26) (Bennett 2005) was used for the numerical handling and diagrammatic presentation of results. Scientific names of vascular plants follow Jørgensen & León-Yánez (1999) except for *Spermacoce remota* Lam. (= taxon formerly identified as *Borreria laevis* and *Diodia radula*; see Tye & Francisco-Ortega 2011). Species authorities for fungal spores are as follows: *Sporormiella*, *Cercophora*, *Podospora*, *Sordaria* and *Coniochaeta* (van Geel & Aptroot 2006), *Delitschia* and *Trichodelitschia* (Cugny 2011), *Fimetariella* N. Lunqvist, *Hypocopra* (Fr.) Kickx, *Petriella* Curzi.

Sample preparation and analyses were conducted in the Long-term Ecology Laboratory, University of Oxford, Oxford, UK and the Institute of Plant Sciences, University of Bern, Bern, Switzerland. Pollen residues and residual sedimentary material for East and Psidium Bog and the El Chato wetlands are stored in the Department of Zoology, University of Oxford; the Pernettya Bog material is stored at the Institute of Plant Sciences, University of Bern. Pollen identification is based on reference material held at both institutions. Modern pollen collections were obtained from fresh samples collected in the Galápagos Islands and from herbarium samples held at the Charles Darwin Research Station, Galápagos, Ecuador and the California Academy of Sciences, San Francisco, California, USA.

The integrity of the sedimentary records is confirmed by the sequential order of radiocarbon dates and the presence of discrete microfossil levels (Table S2, Figs 1, S5, S6), thus alleviating potential concerns of mixing of the record due to bioturbation by tortoises. Rather than a homogenized record that would result from constant mixing, the cores likely represent a ‘running average’ as peaks in pollen and spore abundance may potentially be smoothed as the sediment is compacted, although well-defined peaks are still clearly apparent throughout all three sequences. This has been demonstrated in other depositional environments occupied by large herbivores, for example elephant watering holes in southern Africa (Ekblom & Gillson 2010).

Macrofossil analysis

Sediment cores for macrofossil analysis at East Bog and Pernettya Bog (Table S1) were collected directly adjacent to the primary pollen analysis cores and independently dated (Table S2). Sediment samples (50 cm^3) were taken at 8 cm intervals, following the protocol described in Birks (2001). Detailed methodology is described in Coffey *et al.* (2011). The total number of *Elatine* seed fragments > 125 μm per 50 cm^3 of sediment in each sample was tallied using a Nikon SMZ800 stereomicroscope. The fossil samples were identified using reference material of herbarium specimens held at the Missouri Botanical Garden, St. Louis, Missouri, USA and examination under a scanning electron microscope (SEM). The macrofossils are stored at the University of Oxford.

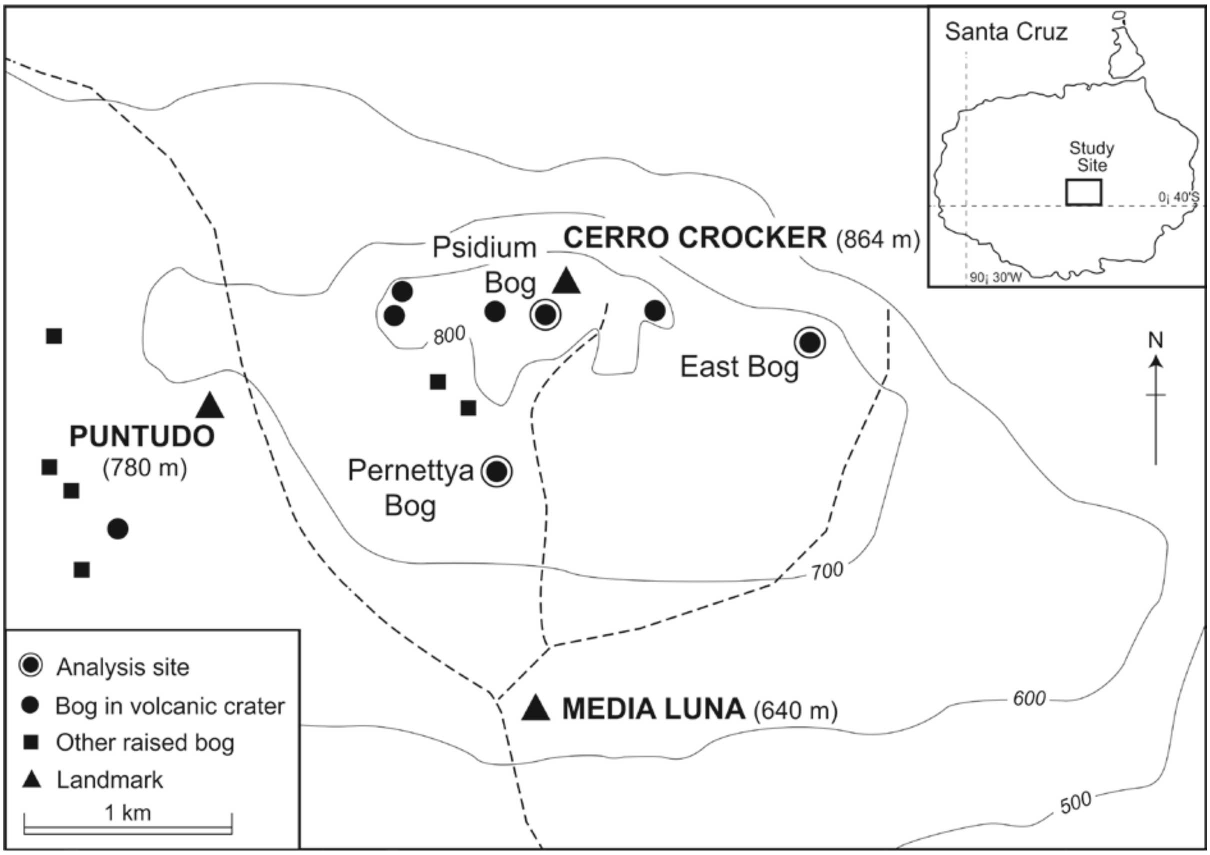

Figure S1 Location of East, Psidium and Pernettya Bog analysis sites described in this study. Location of additional *Sphagnum* bogs present in the Santa Cruz highlands (based on Itow & Weber 1974) are displayed. Circles indicate bogs occurring within volcanic craters; squares, other raised bogs.

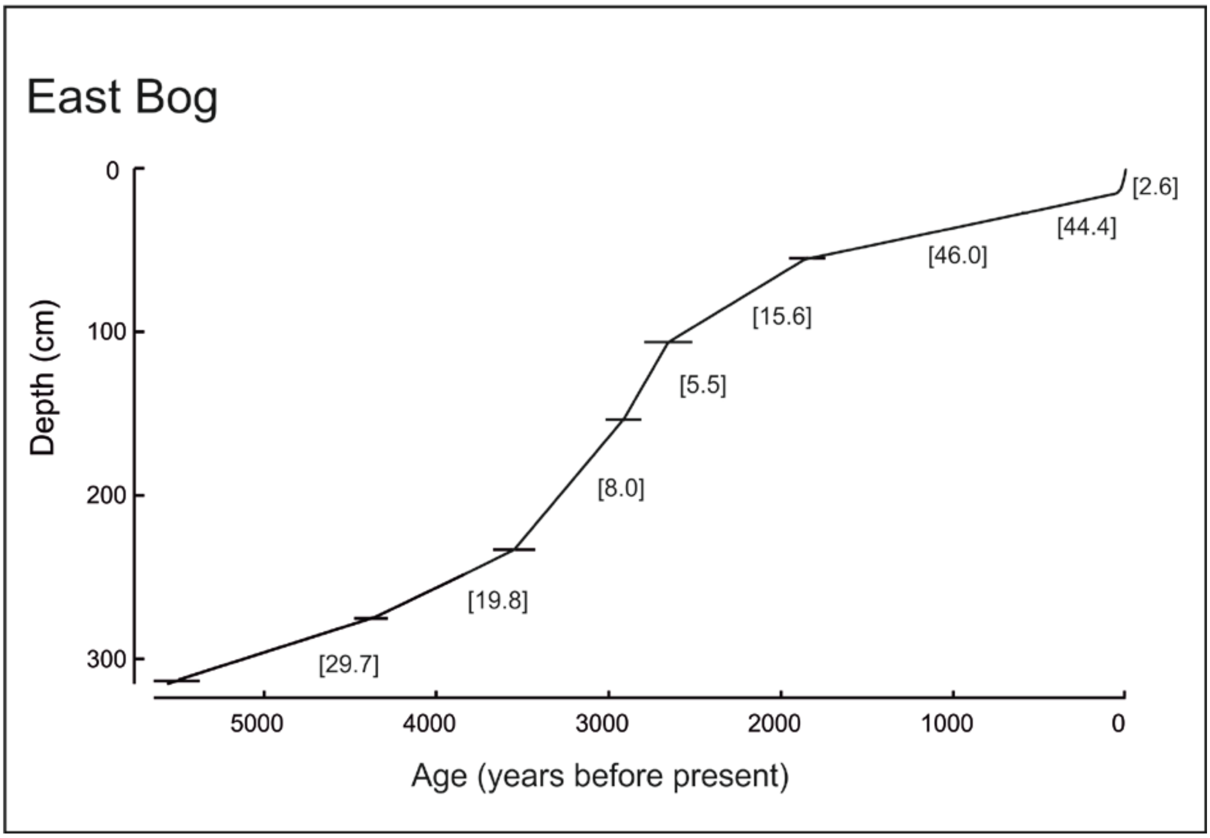

Figure S2 Age–depth model for the East Bog pollen core based on linear interpolation. Ages are displayed as years before present, where Present = AD 2005. Horizontal marks delineate the seven radiocarbon dated sediment samples and standard deviation of the age determinations. Age determinations in the upper 17 cm of the sequence were made using ^{210}Pb dating and the Constant Rate of Supply (CRS) model (Appleby & Oldfield 1978). Sediment accumulation rates (years cm^{-1} of sediment) are indicated in brackets.

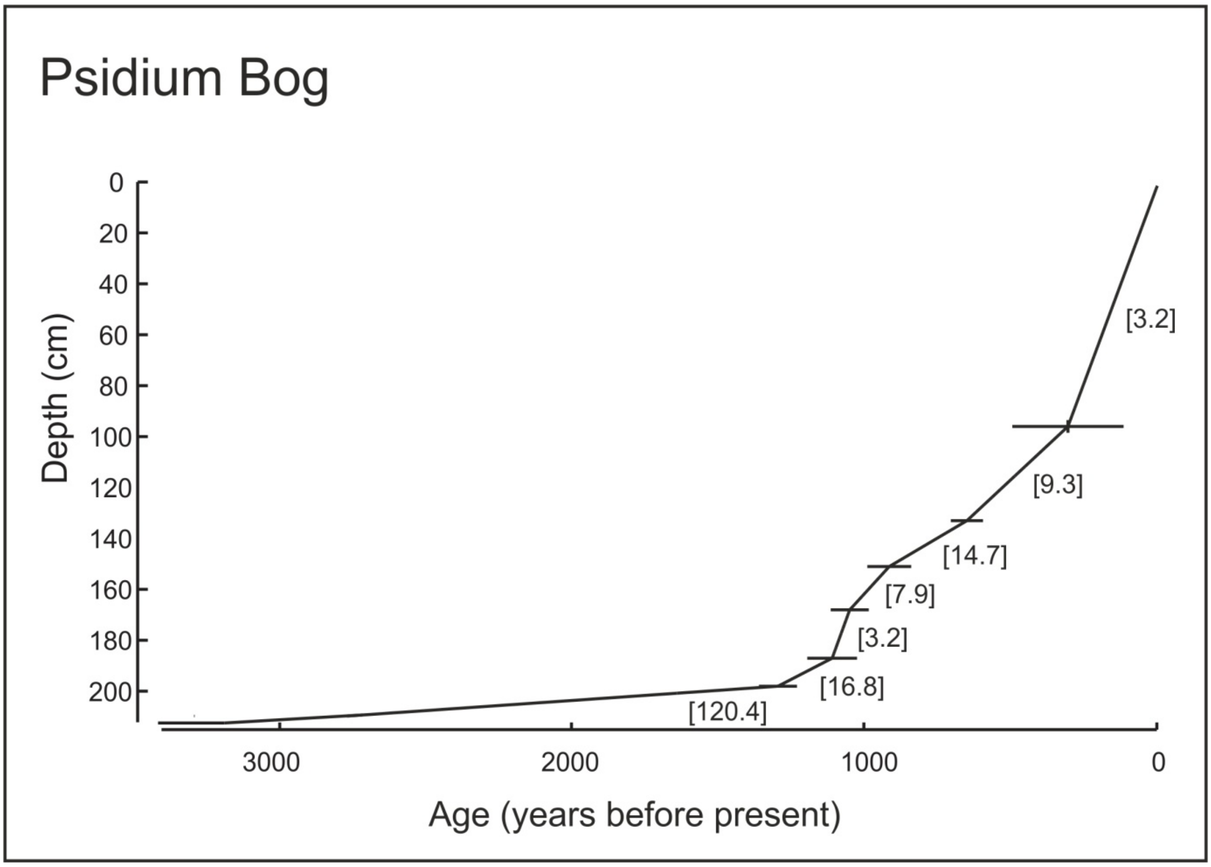

Figure S3 Age–depth model for the Psidium Bog pollen core based on linear interpolation. Ages are displayed as years before present, where Present = AD 2005. Horizontal marks delineate the seven dated sediment samples and standard deviation of the age determinations. Sediment accumulation rates (years cm^{-1} of sediment) are indicated in brackets.

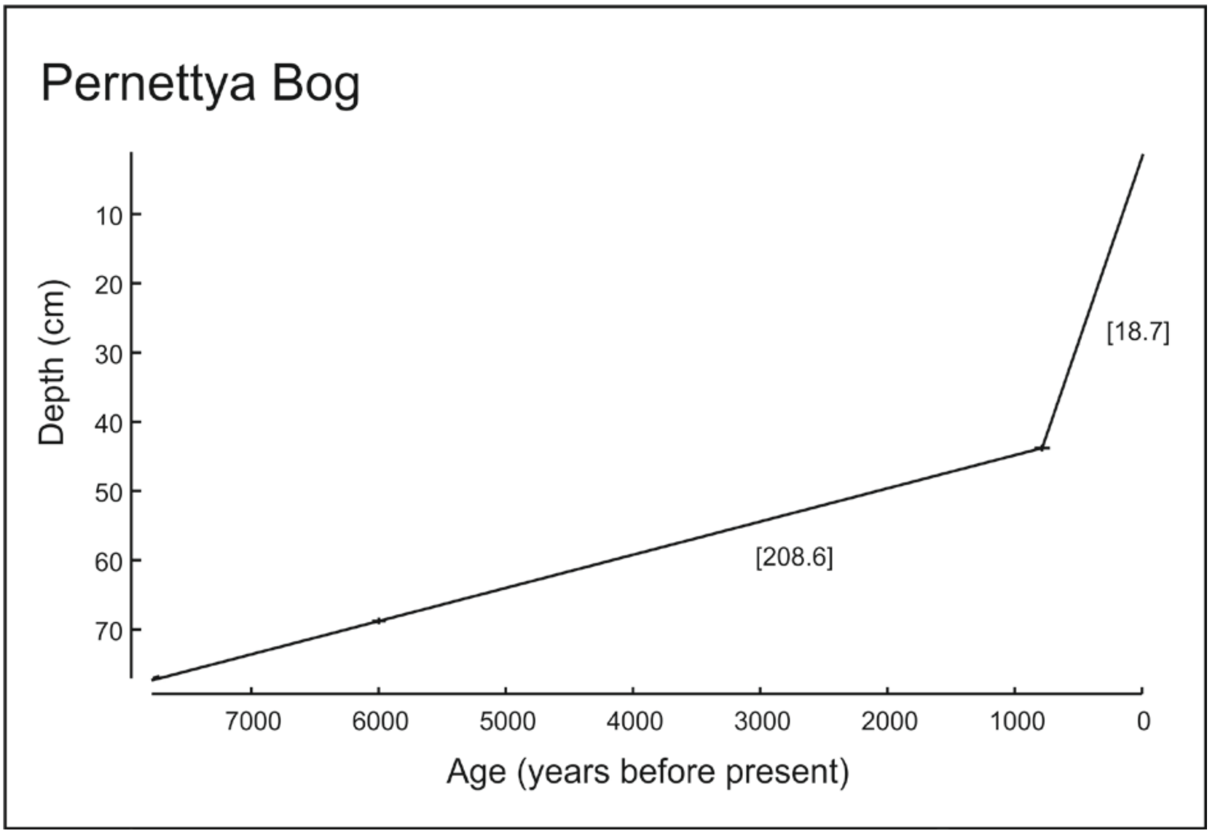

Figure S4 Age–depth model for the Pernettya Bog pollen core based on linear interpolation. Ages are displayed as years before present, where Present = AD 2005. Horizontal marks delineate the two dated sediment samples and standard deviation of the age determinations. Sediment accumulation rates (years cm^{-1} of sediment) are indicated in brackets.

Table S1 Site locations and sedimentary core lengths.

Site	Location	Altitude	Bog Size	Core Length
East Bog (pollen)	S 0º 38' 45", W 90º 19' 03"	739 m	62 × 30 m	344 cm
East Bog (macrofossils)	S 0º 38' 45", W 90º 19' 03"	739 m	62 × 30 m	280 cm
Psidium Bog	S 0º 38' 38", W 90º 19' 37"	809 m	60 × 30 m	326 cm
Pernettya Bog	S 0º 38' 55", W 90º 19' 04"	782 m	19 × 17 m	79 cm
Pernettya Bog (macrofossils)	S 0º 38' 55", W 90º 19' 04"	782 m	19 × 17 m	101 cm

Table S2 Age determinations of sedimentary sequences for pollen and macrofossil analysis. Radiocarbon dates are reported as conventional radiocarbon years BP (AD 1950), analyzed as indicated either by accelerator mass spectrometry (AMS) or conventional dating (gas proportional counting). Calendar ages were calibrated based on the SHCal04 dataset (McCormac *et al.* 2004) calculated at the 2σ level using the probability distribution method, CALIB 5.0.1 (Stuiver & Reimer 1993). Median calendar ages and standard deviations were determined based on a 95% minimum probability of occurrence. Calibrated ages and standard deviations are rounded to the nearest 5-year interval.

Site	Depth (cm)	^{14}C Age (Years BP)	Calendar Age (Cal. yr BP)	Dating Method	Lab Identifier
East Bog (pollen)	27	565 ± 20	530 ± 20	AMS	UBA-15210
	54–55	1910 ± 50	1795 ± 105	Conventional	SWAN-1022
	106	2560 ± 30	2600 ±140	AMS	OxA-17136
	153–154	2810 ± 50	2860 ±105	Conventional	SWAN-1007
	233	3315 ± 50	3495 ± 120	AMS	OZI-800
	275	3945 ± 30	4325 ± 95	AMS	OxA-17235
	313	4790 ± 50	5455 ± 130	Conventional	SWAN-1008
East Bog (macrofossils)	25	420 ± 30	415 ± 90	AMS	UBA-11850
	40	845 ± 30	720 ± 45	AMS	UBA-9484
	112	2695 ± 30	2785 ± 60	AMS	UBA-11851
	131	2835 ± 30	2870 ± 90	AMS	UBA-9485
	277	4375 ± 35	4930 ± 100	AMS	UBA-9486
Psidium Bog (pollen)	92–96	250 ± 50	250 ± 190	Conventional	SWAN-1023
	131	640 ± 35	595 ± 55	AMS	SUERC-24424
	149	1005 ± 35	860 ± 75	AMS	SUERC-24425
	166	1125 ± 35	995 ± 65	AMS	SUERC-24426
	185	1200 ± 30	1055 ± 85	AMS	OZI-803
	196	1390 ± 35	1240 ± 65	AMS	SUERC-24429
	212	3090 ± 35	3250 ± 110	AMS	SUERC-24430
Pernettya Bog (pollen)	43	885 ± 35	740 ± 60	AMS	UBA-8021
	68	5255 ± 30	5955 ± 55	AMS	UBA-8022
Pernettya Bog (macrofossils)	32	Modern*	Modern*	AMS	UBA-16605
	73	6110 ± 30	6900 ± 110	AMS	UBA-16606
	100	8750 ± 35	9640 ± 120	AMS	UBA-16607
El Chato (Core 1)	29-37	Modern*	Modern*	AMS	OZI-801
El Chato (Core 2)	13	Modern*	Modern*	AMS	UBA-13505

* post AD 1950

Table S3 ^{210}Pb dates for the East Bog pollen sequence, analysed using an Ortec HPGe (High Purity Germanium) GWL well-type coaxial germanium detector. Age determinations are based on the Constant Rate of Supply (CRS) model (Appleby & Oldfield 1978) and defined as years before AD 2005.

Site	Depth (cm)	^{210}Pb Age	Method	Lab Identifier
East Bog (pollen)	1	0.6 ± 0.1	CRS Model	Oxford Long-term Ecology Laboratory
	3-4	4.3 ± 0.7	CRS Model	Oxford Long-term Ecology Laboratory
	7-8	11.3 ± 1.9	CRS Model	Oxford Long-term Ecology Laboratory
	11	20.5 ± 3.3	CRS Model	Oxford Long-term Ecology Laboratory
	12-13	27.3 ± 4.6	CRS Model	Oxford Long-term Ecology Laboratory
	15	52.1 ± 8.0	CRS Model	Oxford Long-term Ecology Laboratory

Appendix 2

Pollen and coprophilous fungal spores

Down-washing of coprophilous fungal spores originating from deposition by domestic animals, a potential concern in paleoecological assessments, can be clearly discounted within the Santa Cruz sequences. Firstly, we know that the presence of domestic animals must be limited to post-human discovery (AD 1535). Domestic horses are occasionally present at the sites today and could account for the presence, in small abundance, of spores during the modern period. The pattern of dung spore abundance, however, in all three records is clearly not the result of down-washing of modern material, with little to no presence in the most recent period but high abundance throughout the preceding 5000 years, including clearly defined peaks. Abundance of coprophilous and non-coprophilous fungi, including *Gelasinospora, Ustulina deusta* and *Microthyrium*, was unrelated (Fig. S8), demonstrating that spore abundance is not a by-product of increasing overall fungal activity.

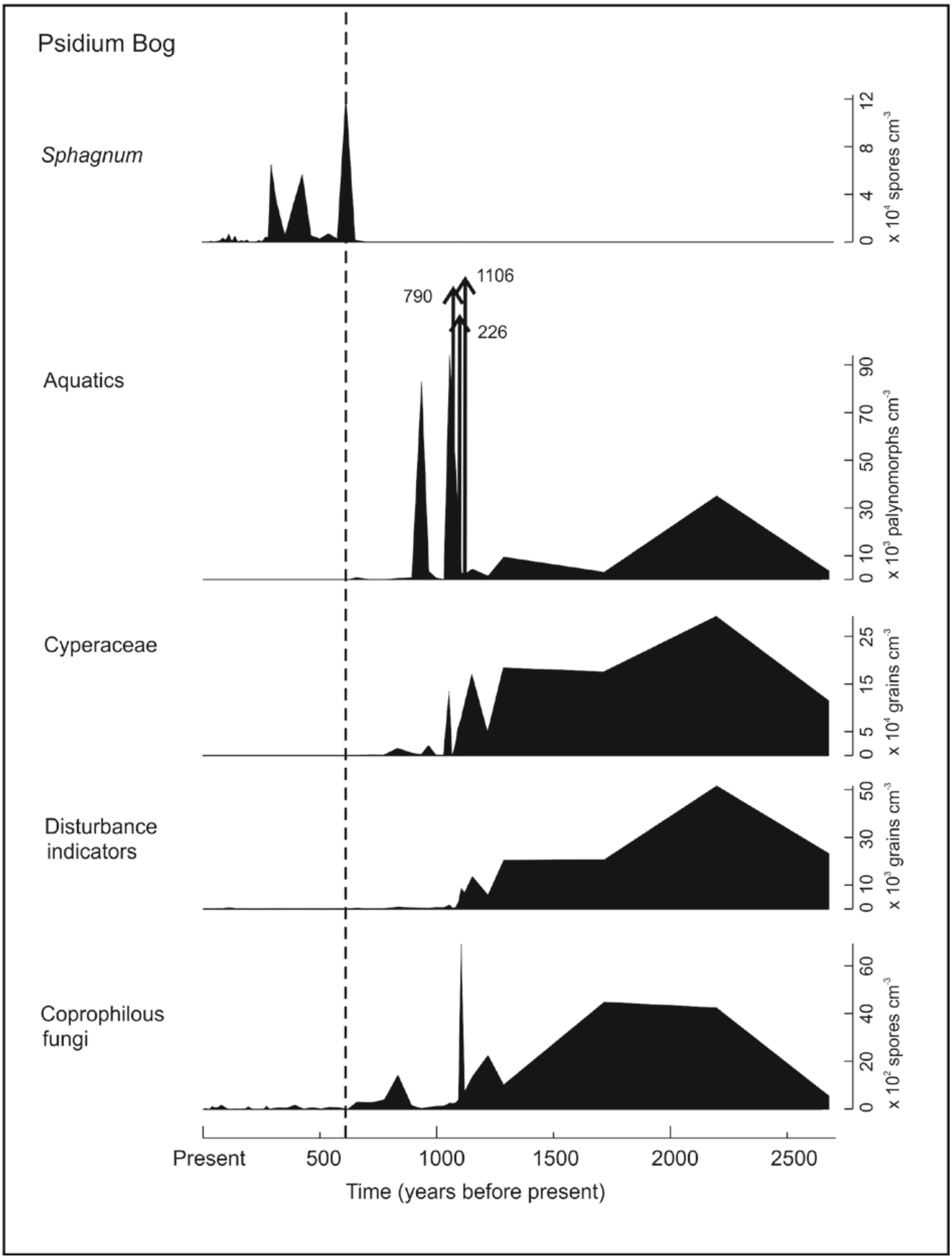

Figure. S5 Variation in concentration (per cm^3 of sediment) of selected pollen and spore types over time at Psidium Bog. Aquatic taxa include: *Utricularia foliosa*, *Azolla microphylla*, *Botryococcus* and *Riccia*. 'Disturbance indicators' are plant species which occupy disturbed, muddy environments and likely indicative of the impacts of tortoise wallowing. These include: *Ageratum conyzoides, Borreria dispersa* species complex, *Spermacoce remota, Commelina diffusa, Cuphea carthagenensis, Drymaria cordata*-type, *Jaegeria gracilis, Ludwigia erecta*-type, *Phyllanthus carolinianus, Polygonum* Sect. *Persicaria* and *Ranunculus flagelliformis.* Coprophilous fungi include: *Sporormiella* and *Cercophora* (2 species).

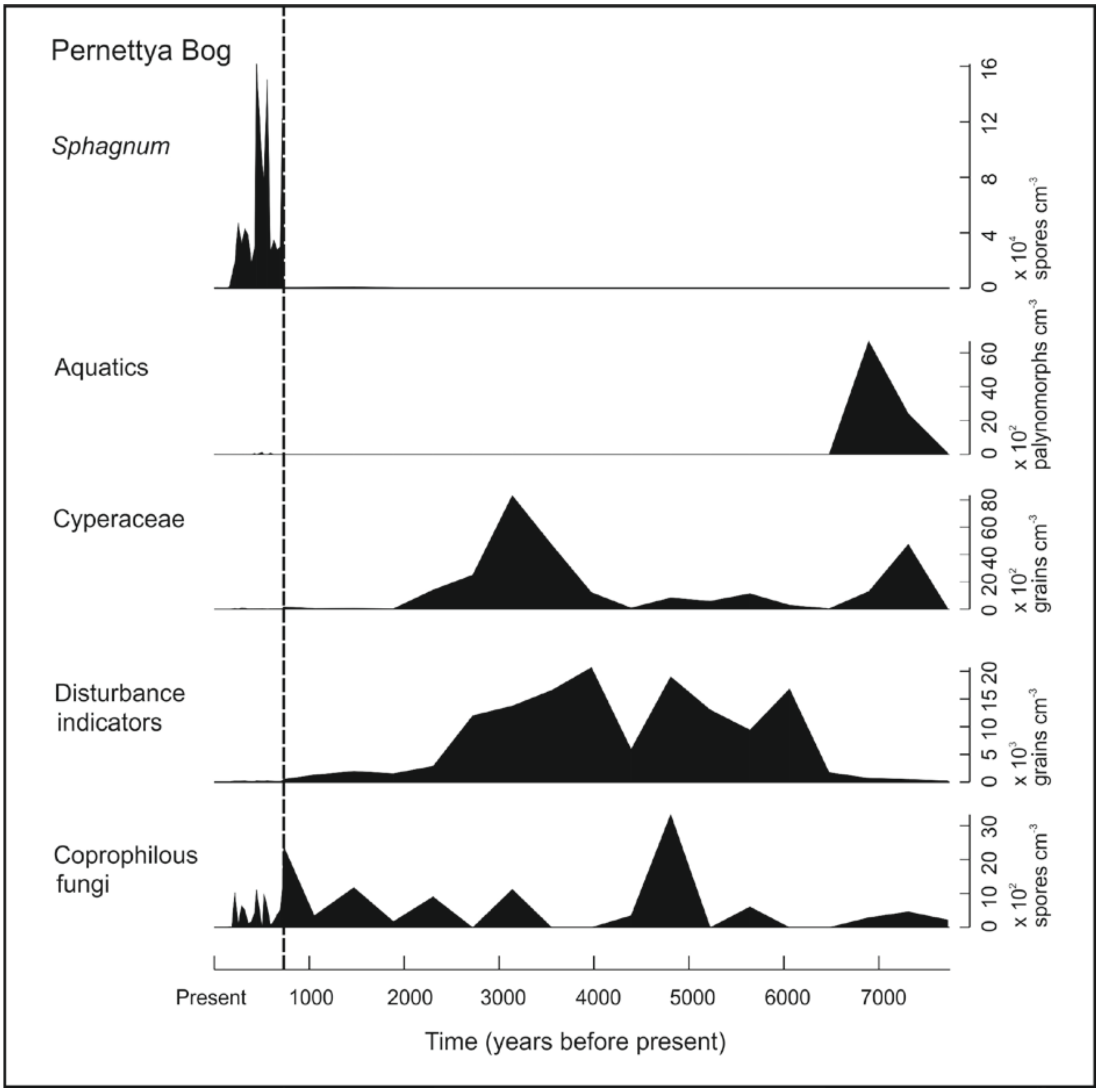

Figure S6 Variation in concentration (per cm^3 of sediment) of selected pollen and spore types over time at Pernettya Bog. Aquatic taxa include: *Azolla microphylla*, *Botryococcus* and *Riccia*. 'Disturbance indicators' are plant species which occupy disturbed, muddy environments and likely indicative of the impacts of tortoise wallowing. These include: *Ageratum conyzoides*, *Borreria dispersa* species complex, *Spermacoce remota*, *Commelina diffusa*, *Cuphea carthagenensis*, *Drymaria cordata*-type, *Jaegeria gracilis*, *Ludwigia erecta*-type, *Phyllanthus carolinianus*, *Polygonum* Sect. *Persicaria* and *Ranunculus flagelliformis*. Coprophilous fungi include: *Sporormiella*, and *Cercophora* (2 species).

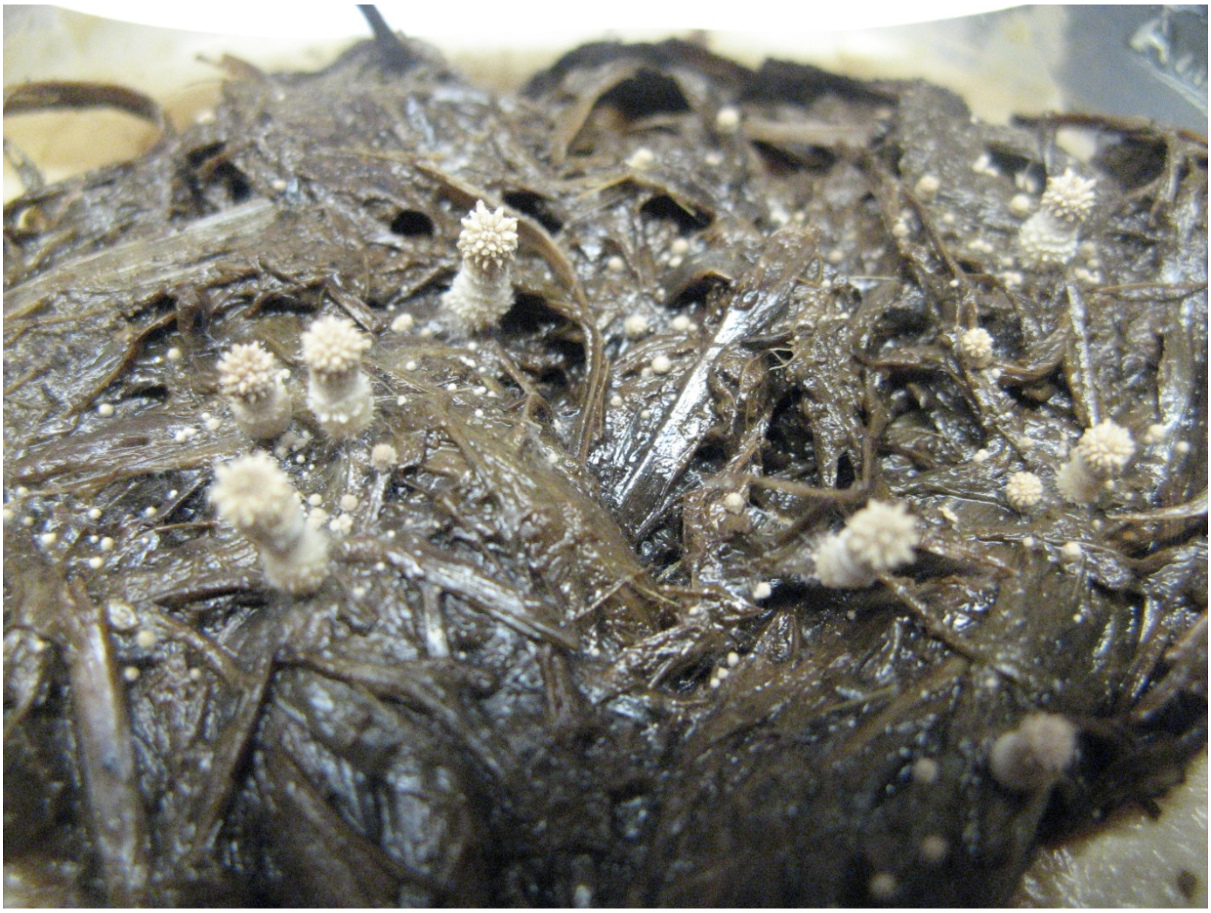

Figure S7 Fungal fruiting bodies growing on fresh giant tortoise dung cultured in the laboratory.

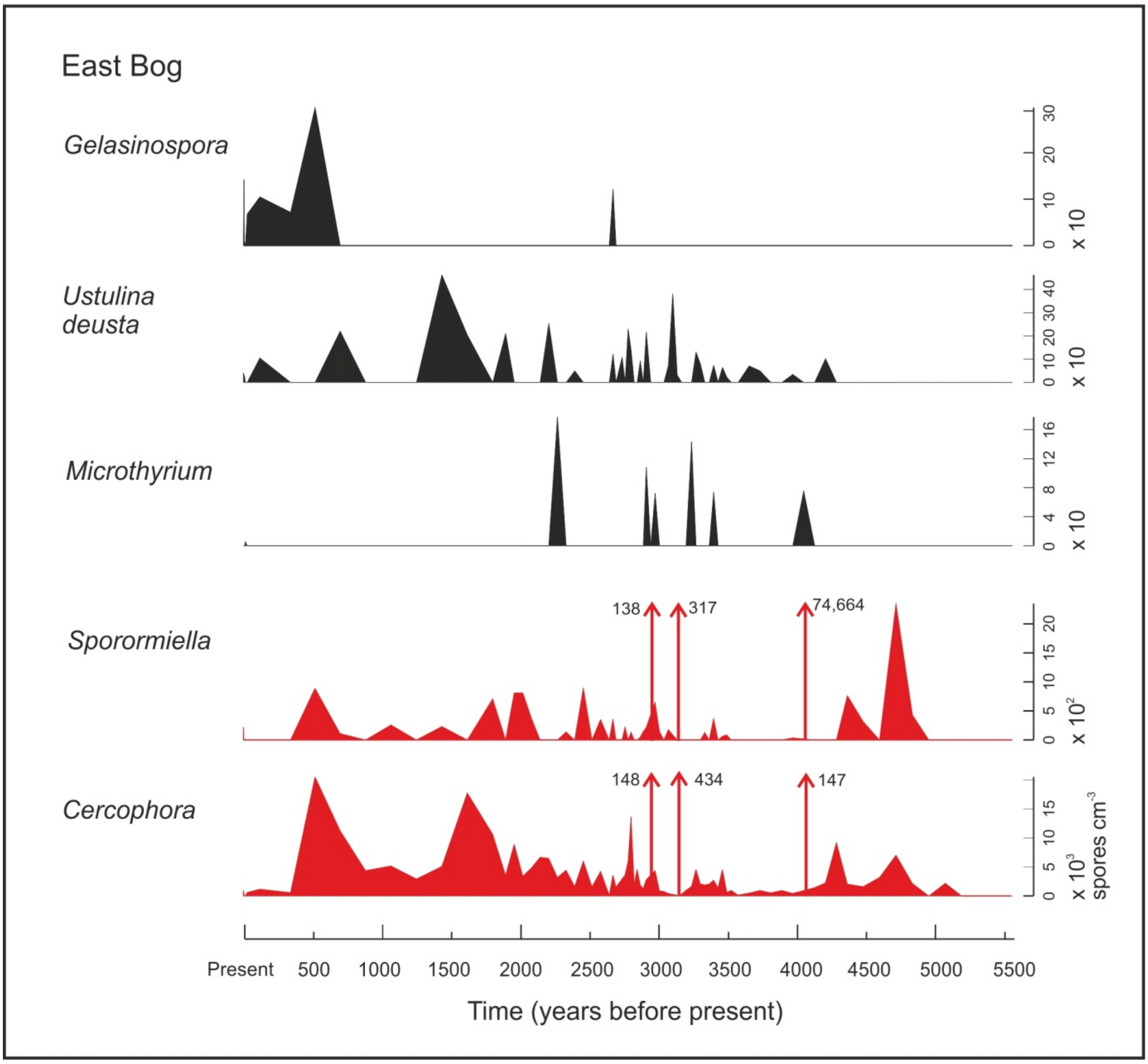

Figure S8 Variation in concentration (per cm^3 of sediment) of abundant fungal spores, both non-coprophilous and coprophilous, over time at East Bog. Non-coprophilous fungi are shown in black, coprophilous fungi in red.

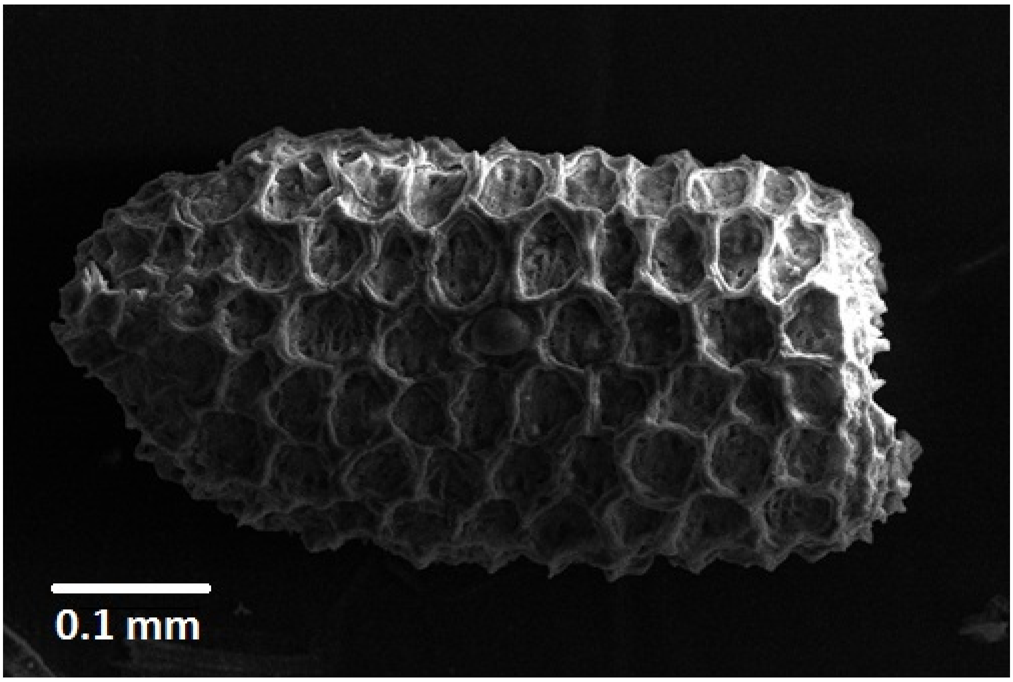

Figure S9 Scanning electron microscope (SEM) image of a fossil seed of *Elatine* sp. from East Bog.

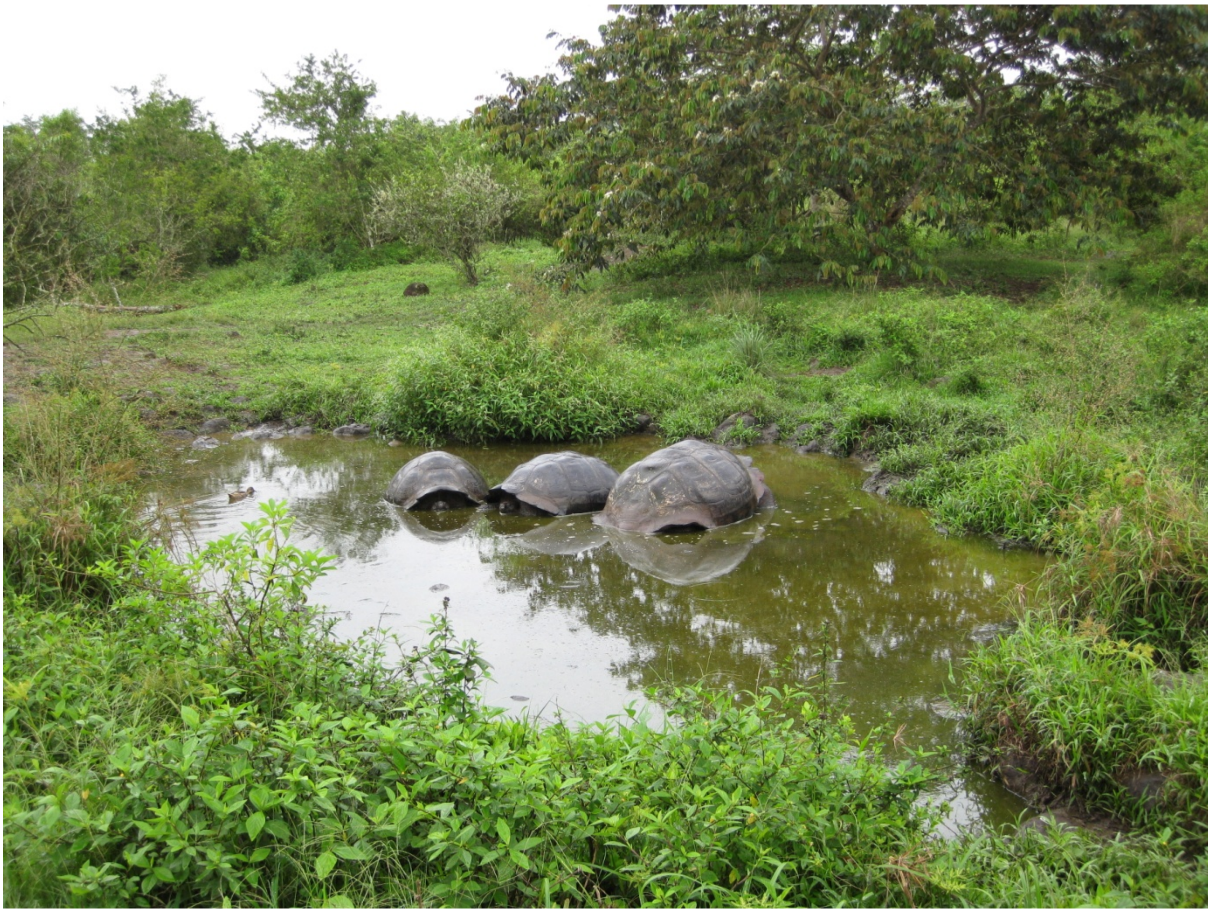

Figure S10 Present-day tortoise habitat at El Chato, Santa Cruz Island. Photo by C.A. Froyd.

Table S4 Number of sampling levels meeting 1%, 2% and 5% *Sporormiella* relative abundance thresholds at each site, excluding singleton spore counts. *Sporormiella* percentages are calculated as a proportion of the sum of *Sporormiella* and the total land pollen (ΣTLP).

Site	1% threshold	2% threshold	5% threshold
East Bog	23	16	4*
Psidium Bog	5	1	1
Pernettya Bog	None		

*Two levels with large spikes in coprophile abundance contained 76% and 99% *Sporormiella*.

Table S5 Avian taxa in the Galápagos (Jiménez-Uzcátegui *et al.* 2012) and their potential to contribute abundant fecal matter to the former upland ponds.

Bahama Pintail (*Anas bahamensis*)	Only resident duck in the archipelago. Prefers coastal lagoons and rarely frequents high elevation sites. Nomadic, solitary species.
Blue-winged Teal (*Anas discors*)	Migrant duck. Uncommon. Not recorded most years and with usually no more than four birds in any one year across the archipelago.
Herons	Galápagos species are coastal and not freshwater-limited. Fairly solitary birds and territorial feeders.
Waders	Migrants, no common species, and no regular freshwater-linked species. Coastal species only, present in relatively low numbers.
Seabirds (gulls, terns, petrels)	Resident species are all coastal. No migrant species.
Pelicans	Only resident species is coastal. No migrant species.
Flamingos	Only resident species is coastal. No migrant species.
Frigates	Present in non-coastal, freshwater habitats but visits are of short duration. Habitat use is only for bathing, do not stay on site.

Supplementary Information References

Appleby P. & Oldfield F. (1978). The calculation of lead-210 dates assuming a constant rate of supply of unsupported lead-210 to the sediment. *Catena*, 5, 1–8.

Bennett K.D. & Willis K.J. (2001). Pollen. In: *Tracking Environmental Change Using Lake Sediments. Volume 3: Terrestrial, Algal, and Siliceous Indicators* (eds. Smol, J.P., Birks, H.J.B. & Last, W.M.). Kluwer Academic Publishers Dordrecht, pp. 5–31.

Bennett K.D. (2009). Psimpoll(4.27). *Psimpoll and pscomb programs for plotting and analysis*. Available at: [*www.chrono.qub.ac.uk/psimpoll/psimpoll.html*]. Last accessed 1 July 2013.

Birks H.H. (2001). Plant macrofossils. In: *Tracking Environmental Change Using Lake Sediments. Volume 3: Terrestrial, Algal, and Siliceous Indicators* (eds. Smol, J.P., Birks, H.J.B. & Last, W.M.). Kluwer Academic Publishers Dordrecht, pp. 49–74.

Coffey E.E.D., Froyd C.A. & Willis K.J. (2011). When is an invasive not an invasive? Macrofossil evidence of doubtful native plant species in the Galápagos Islands. *Ecology*, 92, 805–812.

Cugny C. (2011). *Apports des microfossiles non-polliniques à l'histoire du pastoralisme sur le versant nord Pyrénéen, Entre référentiels actuels et reconstitution du passé.* PhD Dissertation. University of Toulouse-le Mirail, France.

Ekblom A. & Gillson L (2010). Dung fungi as indicators of past herbivore abundance, Kruger and Limpopo National Park. *Palaeogeogr., Palaeoclimatol., Palaeoecol.*, 296, 14–27.

Finsinger W. & Tinner W. (2005). Minimum count sums for charcoal concentration estimates in pollen slides: accuracy and potential errors. *The Holocene*, 15, 293–297.

Jiménez-Uzcátegui G., Wiedenfeld D. A., Vargas F. H., Snell H. L. (2012). CDF Checklist of Galapagos Birds. In: *Charles Darwin Foundation Galapagos Species Checklist*. (eds. Bungartz F., Herrera H., Jaramillo P., Tirado N., Jiménez-Uzcátegui G., Ruiz D., *et al.*). Charles Darwin Foundation, Puerto Ayora, Galapagos. Available at: [http://checklists.datazone.darwinfoundation.org/vertebrates/aves]. Last updated: 22 Nov 2012.

Jørgensen P.M. & León-Yánez S. (1999). *Catalogue of the Vascular Plants of Ecuador*. Missouri Botanical Garden Press, St. Louis MO.

McCormac F.G., Hogg A.G., Blackwell P.G., Buck C.E., Higham T.F.G. & Reimer P.J. (2004). SHCal04 Southern Hemisphere Calibration 0 - 11.0 cal kyr BP. *Radiocarbon*, 46, 1087–1092.

Stockmarr J. (1971). Tablets with spores used in absolute pollen analysis. *Pollen et Spores*, 13, 615–621.

Stuiver M. & Reimer P.J. (1993). Extended ^{14}C database and revised CALIB 3.0 ^{14}C age calibration program. *Radiocarbon*, 35, 215–230.

Tye A. & Francisco-Ortega J. (2011). Origins and evolution of Galápagos endemic vascular plants. In: *The Biology of Island Floras* (eds. Bramwell D. & Caujapé-Castells J.). Cambridge University Press, Cambridge, UK, pp. 89–153.

van Geel B. (2001). Non-Pollen Palynomorphs. In: *Tracking Environmental Change Using Lake Sediments. Volume 3: Terrestrial, Algal, and Siliceous Indicators* (eds. Smol, J.P., Birks, H.J.B. & Last, W.M.). Kluwer Academic Publishers Dordrecht, pp. 99–119.

Van Geel B. (2006). Fossil ascomycetes in Quaternary deposits. *Nova Hedwigia*, 82, 313–329.
